# Cohort Profile: The COVID-19 in Pregnancy in Scotland (COPS) dynamic cohort of pregnant women to assess effects of viral and vaccine exposures on pregnancy

**DOI:** 10.1093/ije/dyab243

**Published:** 2022-01-03

**Authors:** Sarah J Stock, Jade Carruthers, Cheryl Denny, Jack Donaghy, Anna Goulding, Lisa E M Hopcroft, Leanne Hopkins, Rachel Mulholland, Utkarsh Agrawal, Bonnie Auyeung, Srinivasa Vittal Katikireddi, Colin McCowan, Josie Murray, Chris Robertson, Aziz Sheikh, Ting Shi, Colin R Simpson, Eleftheria Vasileiou, Rachael Wood

**Affiliations:** Usher Institute, University of Edinburgh, Edinburgh, UK; Public Health Scotland, Edinburgh, UK; Public Health Scotland, Edinburgh, UK; Public Health Scotland, Edinburgh, UK; Public Health Scotland, Edinburgh, UK; Public Health Scotland, Edinburgh, UK; Public Health Scotland, Edinburgh, UK; Usher Institute, University of Edinburgh, Edinburgh, UK; School of Medicine, University of St Andrews, St Andrews, UK; School of Philosophy, Psychology and Language Sciences, University of Edinburgh, Edinburgh, UK; MRC/CSO Social & Public Health Sciences Unit, University of Glasgow, Glasgow, UK; Institute of Health & Wellbeing, University of Glasgow, Glasgow, UK; School of Medicine, University of St Andrews, St Andrews, UK; Public Health Scotland, Edinburgh, UK; Public Health Scotland, Edinburgh, UK; Department of Mathematics and Statistics, University of Strathclyde, Glasgow, UK; Usher Institute, University of Edinburgh, Edinburgh, UK; Usher Institute, University of Edinburgh, Edinburgh, UK; Usher Institute, University of Edinburgh, Edinburgh, UK; School of Health, Victoria University of Wellington, Wellington, New Zealand; Usher Institute, University of Edinburgh, Edinburgh, UK; Public Health Scotland, Edinburgh, UK

Key Features

- COVID-19 in Pregnancy in Scotland (COPS) is a new national prospective dynamic cohort that was created to describe the epidemiology of COVID-19 in pregnancy and the effect of SARS-CoV-2 infection on pregnancy outcomes and to investigate the safety and effectiveness of COVID-19 vaccines among pregnant women.
- The cohort links primary care records to maternity records, national birth and mortality records and other secondary health care data, together with laboratory results and vaccination information, thus providing a robust platform for the study of viral effects and pharmacoepidemiological research.
- As of 16 September 2021, the dynamic cohort included 123* *004 women with 134* *070 completed or ongoing pregnancies. Pre-pandemic outcome rates for analyses can be calculated from an approved retrospective extension of the cohort to 1 January 2015.
- Data are hosted in the Public Health Scotland’s (PHS) trusted research environment (TRE) and access may be granted via an enquiry to [phs.edris@phs.scot].

## Why was the cohort set up?

Understanding the effects of SARS-CoV-2 infection on maternal, pregnancy and neonatal health is essential to inform public health policy.^1^ The epidemiology of COVID-19 in pregnancy remains incompletely understood as, to date, most studies have included selected cohorts of pregnant women who have required treatment for COVID-19 rather than population-based data.^2–5^ SARS-CoV-2 transmission from mother to baby (antenatally or intrapartum) appears to be possible, but the proportion of pregnancies affected and its clinical significance are uncertain.^6^^,^^7^ Potential effects of the virus on early pregnancy losses, congenital anomalies and fetal growth remain largely un-investigated, and studies have reported conflicting findings on associations between COVID-19 and late miscarriage, preterm birth and stillbirth.^2^^,^^7^

Understanding the effects of COVID-19 in pregnancy and perinatally at different stages will help inform policy and advice to pregnant women and those considering pregnancy, and will provide a platform for studies of long-term effects. It is also essential to inform immunization strategies via assessing the safety and effectiveness of vaccination in pregnancy. Pregnant women have been largely excluded from clinical trials of COVID-19 vaccines, thus despite pregnant women being considered as a vulnerable group, it was initially unclear whether vaccination should be offered to pregnant women,^8–10^ This early lack of clarity regarding vaccine recommendations to pregnant women has contributed to low uptake of COVID-19 vaccination in this group, despite evidence of vaccine effectiveness and safety from observational studies.^8–10^

**Figure 1:**
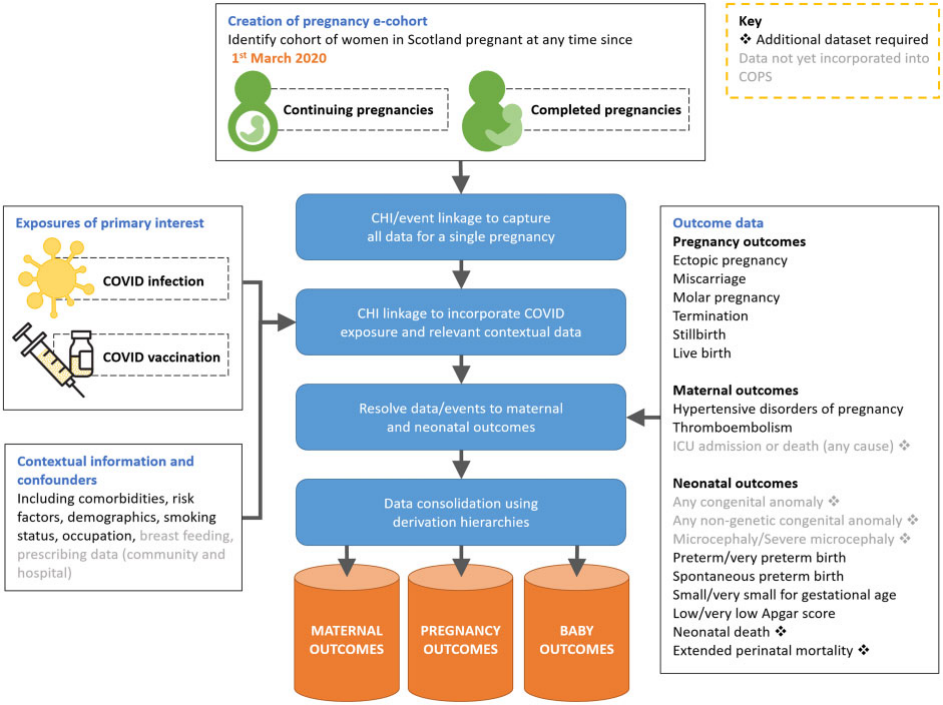
Overview of the creation of the COPS cohort. CHI, Community Health Index; COVID, COVID-19 [coronavirus disease 2019]; ICU, intensive care unit; COPS, COVID-19 in pregnancy in Scotland

COPS^11^ is a sub-study of the EAVE II cohort (Early Pandemic Evaluation and Enhanced Surveillance of COVID-19), an observational study using linked Scottish national data,^12–17^ funded by the Medical Research Council and Scottish Government Director-General for Health and Social Care, Tommy’s Charity and the Wellcome Trust. An overview of the cohort is provided in Figure 1.

## Who is in the cohort?

COPS is a sub-study of EAVE II using unconsented data, which is covered by National Research Ethics Service Committee, South East Scotland 02 approval reference REC 12/SS/0201: SA 2. COPS has been approved by the Public Benefit and Privacy Panel approval reference 2021–0116. Public Health Scotland and the Chief Medical Officer for Scotland are both (independent) data controllers for the national Abortion Act Scotland (AAS) database of termination of pregnancy notifications; thus the Chief Medical Officer has been informed of the use of AAS records for this study and permission to access to the AAS database was granted. All permissions to link the datasets were received by 31 August 2020.

COPS is a national dynamic cohort of all women who were pregnant on, or became pregnant after, 1 March 2020.^11^ Ongoing pregnancies are identified from antenatal booking records. Completed pregnancies are identified from general and maternity hospital discharge records, general practitioner (GP) records, statutory termination of pregnancy records and statutory live and stillbirth registrations (see Table 1 for an overview of data sources; a more detailed description of how the pregnancy cohort was set up is available in Supplementary material, available at *IJE* online). Hospital and GP records capture women who have early pregnancy losses (miscarriage, molar pregnancy or ectopic pregnancy) and receive care from a health care provider. Women who had a very early pregnancy loss and who do not attend or notify their GP or attend hospital for care will not be included. However, we anticipate that these numbers will be small, as, in Scotland: (i) the National Health Service (NHS) provides free health care to all women; (ii) pregnant women are advised to see their GP or attend an early pregnancy unit if they have any signs of a miscarriage; and (iii) clinicians and miscarriage support groups have informed us that only a small minority of women have a miscarriage and do not seek care.^28^^,^^35^^,^^36^ As statutory birth records are used, we capture all births including home births (<2% of births in Scotland), although clinical maternity data may be missing for a proportion of home births. There are no private obstetric services in Scotland.

**Table 1:** Data sources used to generate the COVID-19 in Pregnancy in Scotland (COPS) cohort

Data source	Description
**Identification of pregnant women, associated pregnancy start/end dates, and maternal, pregnancy and neonatal outcomes**	
Antenatal booking records	A national data return developed as part of the response to the COVID-19 pandemic, providing information on all women booking for antenatal care with National Health Service (NHS) maternity services throughout Scotland. More than 99% of women who give birth in Scotland book for antenatal care with NHS maternity services Use: identification of women with ongoing pregnancies in near-real time
General practioner (GP) record data	A bespoke data feed from all GP practices in Scotland containing information on women who have miscarriage, ectopic pregnancy and molar pregnancy Use: identification of women with early miscarriage, molar pregnancy or ectopic pregnancy not admitted to hospital (i.e. those cared for by their GP and those receiving outpatient/day patient care in a hospital setting such as an early pregnancy centre)
Scottish Morbidity Record (SMR) 01^18^	The SMR01 database includes all general day case and inpatient admissions in Scotland. Admissions to neonatal, maternity and mental health care are excluded from SMR01 as they are covered by other specialist datasets Use: identification of women with early miscarriage, molar pregnancy or ectopic pregnancy admitted to hospital
Abortion Act Scotland (AAS) records^19^	Statutory notifications of termination of pregnancy, including those indicated by congenital anomaly Use: identification of women who have termination of pregnancy
Scottish Morbidity Record (SMR) 02^20^	The SMR02 database includes all day case and inpatient admissions to maternity specialties in Scotland Use: identification of miscarriage, stillbirth and live births managed in hospital (≥98% of births in Scotland) and some home births (≤2% of births in Scotland), some ectopic, molar and terminations of pregnancy cared for in maternity settings
National Records of Scotland (NRS) statutory stillbirth registrations^21^	Scottish legislation requires all stillbirths at 24 weeks of gestation or more to be registered with NRS within 21 days of birth Use: identification of stillbirths
National Records of Scotland (NRS) statutory live birth registrations^22^	Scottish legislation requires all live births at any gestation to be registered with NRS within 21 days of birth Use: identification of live births
NHS Live Births	New national data return developed as part of the response to the COVID-19 pandemic, providing information on live births notified by maternity services to NHS Board child health administrative departments: for near-real time access to data, which allows intergenerational linkage of records relating to mothers and their babies if statutory live birth registration is suspended Use: identification of livebirths
Scottish Intensive Care Society Audit Group (SICSAG) records^23^	National database of patients admitted to adult general critical care units in Scotland, detailing information on the management of critically ill or injured patients. All general intensive care units and combined ICU/high dependency units (HDU) collect data and more than 90% of general high dependency units and a number of specialist ICU and HDUs also provide records Use: identification of women admitted to intensive care
Scottish Birth Record (SBR)^24^	The SBR records basic demographic data on all births in Scotland and additional clinical information and diagnostic and operational procedure codes on babies admitted to neonatal care Use: identification of neonates admitted to neonatal care
Scottish linked congenital anomaly database^25^	National database of congenital anomalies with data derived from SMR02, SMR01, AAS, SBR, NRS statutory stillbirth and death registration and Mothers and Babies: Reducing Risk through Audits and Confidential Enquiries across the UK (MBRRACE-UK) records Use: identification of babies with congenital anomalies and classification of congenital anomaly
**Identification of women with confirmed or suspected COVID-19**	
Electronic Communication of Surveillance in Scotland (ECOSS)^26^ and other virology results held separately by Public Health Scotland (PHS)	ECOSS is a database that holds surveillance data on various microorganisms (e.g. influenza virus, coronavirus) and infections reported from NHS diagnostic and reference laboratories and Pillar 2 facilities/Lighthouse laboratories [high-throughput facilities dedicated to COVID-19 viral Reverse Transcription-Polymerase Chain Reaction (RT-PCR) testing for the National Testing Programme]. Data on laboratory results for all SARS-CoV-2 RT-PCR tests carried out in Scotland are being collated by ECOSS and can be linked to other data sources Use: identification of women and neonates with viral RT-PCR tests for SARS-CoV-2
National Records of Scotland (NRS) statutory death registrations^27^	National statutory death records Use: identification of women with COVID-19 recorded as cause of death
SMR01^18^, SMR02^20^ and NRS stillbirths^21^	As described above Use: identification of women with COVID-19 recorded as cause of admission/stillbirth
**Identification of treatments, vaccination status and risk group**	
Early Pandemic Evaluation and Enhanced Surveillance of COVID-19 (EAVE II) cohort	An extract of current and past diagnoses from the EAVEII cohort,^28^ which is based on all 5.4 million individuals registered with a GP in Scotland from 23 February 2020 (98–99% of the Scottish population) Use: identification of comorbidities and COVID-19 risk grouping (using the QCOVID^29^ risk grouping plus hypertension)
Shielding list	Public Health Scotland list drawn from a number of data sources including those thought to be extremely clinically vulnerable Use: identification of extremely clinically vulnerable pregnant women
Vaccine Management Tool (VMT) records^30^	The Turas VMT is a new application that has been developed by NHS Education for Scotland to record delivery of COVID-19 vaccination in different NHS and community settings Use: identification of vaccination and vaccination date
GP vaccination data	An extract from GP records with data on vaccinations administered in practices that are not using the VMT Use: identification of vaccination and vaccination date
Health care worker records: Scottish Workforce Information Standard System (SWISS+)^31^	A new national health care worker (HCW) dataset to support analyses relating to COVID-19 in this occupational group. The HCW data within the dataset are derived from an extract of the SWISS system with information of staff directly employed by the NHS and GPs contracted to provide NHS care Use: identification of women eligible for vaccination due to health care worker status
Child Health Systems Program (CHSP) – Pre-school (PS)^32^	The CHSP-PS system supports the delivery of the child health programme by facilitating the automated call and recall of children for the agreed schedule of child health reviews for pre-school children Use: infant feeding records to identify women vaccinated during breastfeeding
**Additional data sets with governance approval for future linkage**	
Serology databases	Residual sera from blood tests taken for combined first trimester screening for fetal trisomies such as Down’s syndrome, offered as part of routine antenatal care, are being tested for SARS-CoV-2 antibodies as part of the surveillance of the pandemic in Scotland Proposed use: identification of women with serological evidence of infection and vaccination
Community prescribing [PIS] records^33^	This includes information on all prescribed medications that are dispensed in the community in Scotland Proposed use: identification of comorbidities and COVID-19 treatments given
Scottish Hospital Electronic Prescribing and Medicines Administration (HEPMA)^34^	Electronic records of hospital-administered treatments, currently available within four Scottish hospitals Proposed use: identification of COVID-19 treatments given

The cohort is updated monthly, allowing near ‘real-time’ identification of pregnant and recently pregnant women. There are differences in source data latency to the unified COPS dataset (see Supplementary material), which means that data for the most recent months are most unstable, with potential for missing conceptions and end-of-pregnancy events; initial findings may be ‘overruled’ over time as more detailed records accrue. Data are generally complete for conceptions and end-of-pregnancy events occurring up to 3–4 months previously.

As of 16 September 2021, the cohort included 123* *004 women with 134* *070 completed or ongoing pregnancies. We have completed pregnancy outcomes for the first wave COVID-19 cohort, which included women who were pregnant on the 1 March 2020 and those who became pregnant up to 30 June 2020 (*n* = 60* *402 pregnancies). The cohort continues to be updated and the end date depends on the course of the pandemic and requirement to support future pandemic preparedness.

## How often have they been followed up?

Data are collected from women throughout their pregnancy to 41 days postpartum, and data on their babies are collected up to the end of the neonatal period (27 days after birth).

Women enter the cohort on identification of a pregnancy from one or more sources of routinely collected health care data from primary and secondary care settings (see Table 1). Pregnancy outcome (i.e. ectopic pregnancy, molar pregnancy, miscarriage, termination of pregnancy, stillbirth or live birth) is obtained from the same datasets, and data are linked to a number of other sources (also summarized in Table 1) to investigate maternal demographics and comorbidities. Further information on fetal and neonatal outcomes [congenital anomaly, preterm birth, very preterm birth, small for gestational age, severe small for gestational age, microcephaly, severe microcephaly, low Apgar score, very low Apgar score, neonatal SARS-CoV-2 infection (see Supplementary Table S1, available as Supplementary data at *IJE* online, for definitions), neonatal mortality and extended perinatal mortality)] and maternal outcomes [COVID-19 disease, severe COVID-19 disease (see Supplementary Table S1 for definitions), any maternal death, thromboembolic disease, hypertensive disorders of pregnancy] also come from the same datasets.

New pregnant women are added to the cohort and new outcomes are identified at monthly updates. Women who have given birth, and their babies, remain in the cohort. Pregnant women who leave Scotland before pregnancy end will have the pregnancy outcome recorded as unknown, but will remain in the cohort. Linkage through a universal health care identifier (Community Healthcare Index or CHI) will allow further follow-up of women and children unless they leave Scotland permanently.

## What has been measured?

The data sources provide comprehensive information on pregnancy and maternal and neonatal outcomes, as well as complications, pre-existing and pregnancy risk factors, clinical vulnerability to COVID-19, COVID-19 diagnosis and vaccination status. An overview of the maternal and pregnancy characteristics and pregnancy and neonatal outcome data that are collected are in Table 2. A high-level summary of other key exposure and outcome data being collected is provided in Supplementary Table S1. Detailed description and definitions of outcomes can be found in the COPS data dictionary.^37^

**Table 2:** Maternal characteristics and key pregnancy and neonatal outcomes of the dynamic COVID-19 in Pregnancy in Scotland (COPS) (up to mid-September 2021) and first wave cohort (women who were pregnant on 1 March 2020 or who became pregnant between 1 March 2020 and 30 June 2020)

	COPS dynamic cohort (as of 16 September 2021)		COPS COVID-19 first wave cohort (pregnancies from 1 March to 30 June 2020)	
Number of women	123* *004		59* *926	
Number of pregnancies	134* *070		60* *402	
Number of liveborn babies	71* *684		48* *910	

	*n*	% of pregnancies	*n*	% of pregnancies

Age at conception				
≤19 years	6331	4.7	2782	4.6
20–24 years	20* *742	15.5	9400	15.6
25–29 years	36* *956	27.6	17* *037	28.2
30–34 years	41* *749	31.1	18* *964	31.4
35–39 years	22* *675	16.9	9948	16.5
40–44 years	4997	3.7	2070	3.4
≥45 years	350	0.3	140	0.2
Unknown^1^	270	0.2	61	0.1
Deprivation level (Scottish Index of Multiple Deprivation (SIMD) quintile)				
1 (most deprived)	32* *514	24.3	14* *593	24.2
2	27* *800	20.7	12* *406	20.5
3	24* *185	18.0	10* *925	18.1
4	26* *580	19.8	12* *156	20.1
5 (least deprived)	22* *128	16.5	10* *066	16.7
Unknown	863	0.6	256	0.4
Self-reported ethnicity				
Black/Caribbean/African	1801	1.3	921	1.5
Chinese	636	0.5	341	0.6
Mixed or other ethnic group	3894	2.9	1977	3.3
South Asian	3903	2.9	1970	3.3
White	94* *230	70.3	47* *199	78.1
Unknown	29* *606	22.1	7994	13.2
NHS Board of Residence				
Ayrshire and Arran	8108	6.0	3658	6.1
Borders	2194	1.6	1015	1.7
Dumfries and Galloway	3004	2.2	1431	2.4
Fife	8812	6.6	3956	6.5
Forth Valley	7290	5.4	3275	5.4
Grampian	14* *256	10.6	6465	10.7
Greater Glasgow and Clyde	29* *965	22.4	13* *618	22.5
Highland	6621	4.9	3022	5.0
Lanarkshire	17* *678	13.2	7784	12.9
Lothian	23* *983	17.9	10* *833	17.9
Orkney	448	0.3	212	0.4
Shetland	479	0.4	223	0.4
Tayside	9831	7.3	4425	7.3
Western Isles	468	0.3	209	0.3
Outside Scotland	18	0.0	2	0.0
Unknown	915	0.7	274	0.5
Maternal urban/rural classification				
Very remote rural areas	1642	1.4	903	1.5
Very remote small towns	868	0.8	466	0.8
Remote rural areas	2160	1.9	1168	1.9
Remote small towns	2142	1.9	1164	1.9
Accessible rural areas	10* *288	9.0	5593	9.2
Accessible small towns	7564	6.6	4038	6.7
Other urban areas	34* *259	29.8	18* *281	30.2
Large urban areas	38* *724	33.7	20* *487	33.8
Unknown	17* *249	15.0	8490	14.0
Maternal body mass index (BMI; kg/m^2^) at pregnancy booking or pre-pregnancy				
<18.5	3092	2.3	1587	2.6
18.5–<25	39* *113	29.2	20* *697	34.3
25–<30	29* *731	22.2	15* *920	26.4
30–≤40	22* *803	17.0	12* *231	20.2
≥40	4242	3.2	2223	3.7
Unknown	35* *089	26.2	7744	12.8
Smoking status at pregnancy booking				
Current smoker	13* *272	9.9	6706	11.1
Former smoker	15* *205	11.3	7450	12.3
Never smoker	77* *537	57.8	37* *225	61.6
Unknown	28* *056	20.9	9021	14.9
Clinical vulnerability group				
Clinically extremely vulnerable risk group status^2^	1119	0.8	572	0.9
Clinically vulnerable risk group status^3^	35* *364	26.4	15* *996	26.5
No clinical vulnerable group identified	97* *587	72.8	43* *834	72.6
Plurality				
Singleton pregnancy	71* *828	53.6	48* *365	80.1
Multiple pregnancy	1078	0.8	709	1.2
Unknown	61* *164	45.6	11* *328	18.8
Gestation at end of pregnancy				
≤12 weeks	28* *909	21.6	8848	14.6
13–23 weeks	2787	2.1	1144	1.9
24–27 weeks	396	0.3	210	0.3
28–31 weeks	615	0.5	358	0.6
32–36 weeks	4416	3.3	2790	4.6
37–41 weeks	63* *999	47.7	43* *959	72.8
≥42 weeks	4421	3.3	3093	5.1
Pregnancy ongoing	28527	21.3	0	–

	*n*	% of completed pregnancies (*n* = 105* *543)	*n*	% of completed pregnancies

Pregnancy outcome				
Miscarriage^4^	11* *733	11.1	3563	5.9
Ectopic pregnancy	1108	1.0	300	0.5
Termination	18* *533	17.6	6013	10.0
Stillbirth	243	0.2	158	0.3
Live birth	70* *538	66.8	48* *151	79.7
Unknown	3388	27.0	2406	4.0
Neonatal outcomes^5^	*n*	% of live born babies	*n*	% of live born babies

Sex of baby				
Male	36* *767	51.3	25* *074	51.3
Female	34* *917	48.7	23* *836	48.7
Birthweight				
<1000 g	249	0.3	117	0.2
1000–1499 g	369	0.5	220	0.4
1500–2499 g	3620	5.0	2533	5.2
2500–4499 g	59533	83.0	43* *751	89.5
>4500 g	1190	1.7	870	1.8
Unknown	6723	9.4	1419	2.9
Neonatal death				
Early neonatal death (0–6 days)	101	0.14	58	0.12
Late neonatal death (7–27 days)	49	0.07	28	0.06
Survived neonatal period	71* *534	99.8	48* *824	99.8

1 Age <10 or ≥55 classified as unknown.

2 People with one or more of the following conditions indicate inclusion on the Scottish shielding list: solid organ transplant, specific cancers, severe respiratory conditions including all cystic fibrosis, severe asthma and severe chronic obstructive pulmonary disease (COPD), rare diseases and inborn errors of metabolism that significantly increase the risk of infections (such as severe combined immunodeficiency [SCID], homozygous sickle cell disease), immunosuppression therapies sufficient to significantly increase risk of infection, pregnant with significant heart disease, congenital or acquired, other clinical indication.

3 People with one or more of the following comorbidities that are associated with severe COVID-19 outcomes in the general adult population (based on Q-COVID^29^) and/or pregnant women specifically^3^ and are reasonably prevalent (>0.5%) among women of reproductive age in Scotland (as identified using EAVE II data^17^): asthma, congenital heart disease, renal failure [chronic kidney disease (CKD)3, CKD4, CKD5, with or without dialysis or transplant], epilepsy, type 1 diabetes, type 2 diabetes, rheumatoid arthritis or systemic lupus erythematosus, venous thromboembolism, severe mental illness, body mass index (BMI) ≥40 kg/m^2^, hypertension (based on read coded diagnosis within general practioner record), any other comorbidity included in the Q-COVID algorithm that is available through the EAVE II GP dataset [chronic obstructive pulmonary disease, rare respiratory conditions (cystic fibrosis, bronchiectasis or alveolitis), pulmonary hypertension or pulmonary fibrosis, coronary heart disease, atrial fibrillation, heart failure, stroke, peripheral vascular disease, cirrhosis, cerebral palsy, Parkinson’s disease, rare neurological conditions (motor neurone disease, multiple sclerosis, myasthenia, Huntington’s chorea), dementia, blood cancer, lung or oral cancer, sickle cell disease, osteoporotic fracture]

4 Includes molar pregnancy.

5 The following neonatal characteristics are also available: SIMD deprivation quintile; ethnicity; NHS Board Of Residence; urban/rural classification.

## What has it found?

There were 38* *106 ongoing pregnancies in Scotland on 1 March 2020. As of the 16 September 2021, data on a further 95* *964 pregnancies conceived from 1 March 2020 onwards have been added to the cohort. The COVID-19 first wave cohort, with women who were pregnant on 1 March 2020 or who subsequently became pregnant up until 30 June 2020, includes a total of 60* *402 pregnancies.

**Figure 2:**
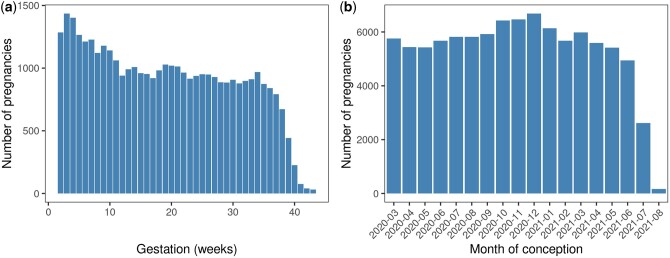
Gestational age of pregnant women on 1 March 2020 (a) and conceptions each month from 1 March 2020 onwards (b)

**Figure 3:**
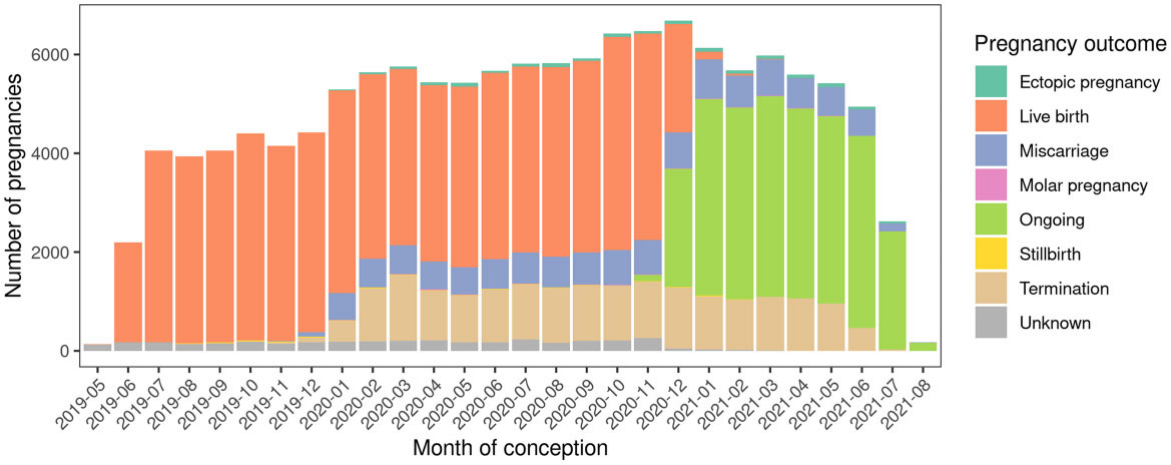
Pregnancy outcomes of the dynamic cohort by month of conception as of mid-September 2021

Figure 2 shows gestational age distribution of ongoing pregnancies on 1 March 2020 (panel a) and the conceptions each month from 1 March 2020 onwards (panel b). Figure 3 shows the outcomes of all pregnancies in the dynamic cohort by month of conception. Maternal characteristics, pregnancy and selected neonatal outcomes of the participants in the dynamic cohort to 16 September 2021 and the COVID-19 first wave cohort are shown in Table 2.

The COPS cohort has been linked to COVID-19 vaccination data which show the changing pattern of uptake of COVID-19 vaccinations by pregnant women.^38^

## What are the main strengths and weaknesses?

To our knowledge, this is the first near complete population-level platform capturing all pregnancy outcomes from conception to the end of the puerperium and neonatal period. Linkage of GP records to established birth records and secondary care records has allowed early pregnancy losses to be included. This linkage has enriched the cohort by identifying 4208 early pregnancy outcomes that are not captured in secondary care records. Linkage to other datasets allows capture of rich data on maternal demographics and comorbidities, COVID-19 disease and COVID-19 vaccination. The potential for future linkage to long-term child and maternal health care data and primary and secondary care prescription data enables a robust platform for future pharmacoepidemiology studies.

Weaknesses of the cohort include the fact that women with early pregnancy loss who do not seek medical advice will not be included. In addition, clinical maternity data on a proportion of home births (which make up <2% of births overall in Scotland) will not be available, although the births themselves will be included. Although a key strength is that we have population-based data on all women with confirmed or probable COVID-19 in pregnancy, and are not restricted to women and babies admitted to hospital, we acknowledge that restriction of viral PCR testing early in the pandemic to health care workers and patients ill enough to require hospital admission, may have limited ascertainment of all cases at that time.^39^^,^^40^

## Can I get hold of the data? Where can I find out more?

The data underlying this article cannot be shared publicly as they are sensitive. Public Health Scotland and the Chief Medical Officer for Scotland are the data holders for the data used in this study. Data are available to researchers for analysis after securing relevant permissions from the data holders. Enquiries regarding data availability should be directed to [phs.edris@phs.scot].

## Author Contributions

S.J.S., R.W., C.R. and A.S. conceived the study. S.J.S., D.M., E.V., C.R.S., U.A., C.M., J.D., L.R., C.R., A.S., L.H., L.E.M.H., A.G., J..C and R.W. designed the study. S.J.S., D.M., E.V., C.R.S., U.A., C.M., L.H., J.D., L.R., C.R., A.S., R.M., S.V.K. and R.W. drafted the protocol. J.D., A.G., C.D., L.H., L.E.M.H. and J.C. performed data analysis. S.J.S., D.M., E.V., C.R.S., U.A., C.M., L.H., L.R., C.R., A.S., A.G., C.D., L.E.M.H., J.C., B.A. and R.W. interpreted the data and revised the manuscript for important intellectual content. S.J.S., D.M., E.V., C.R.S., U.A., C.M., L.H., J.D., L.R., C.R., A.S., A.G., C.D., L.E.M.H., J.C., B.A., S.V.K. and R.W. gave final approval of the version to be published. R.W. acts as guarantor for the study.

## Supplementary Data

Supplementary data are available at *IJE* online.

## Funding

EAVE II is funded by the Medical Research Council (MR/R008345/1) with the support of BREATHE—the Health Data Research Hub for Respiratory Health [MC_PC_19004] which is funded through the UK Research and Innovation Industrial Strategy Challenge Fund and delivered through Health Data Research UK. Additional EAVE II support has been provided through the Scottish Government DG Health and Social Care. COPS receive additional funding from Tommy’s Charity (Charity number 1060508; SC039280) and is supported by Sands (Charity number 299679). S.J.S. is supported by the Wellcome Trust (209560/Z/17/Z). S.V.K. acknowledges funding from an NRS Senior Clinical Fellowship (SCAF/15/02), the Medical Research Council (MC_UU_00022/2) and the Scottish Government Chief Scientist Office (SPHSU17). B.A. was supported by the European Union’s Horizon 2020 research and innovation programme under the Marie Skłodowska-Curie grant agreement No.813546, the Baily Thomas Charitable Fund, the Data Driven Innovation and the UK Economic and Social Research Council (ES/N018877/1) during the course of this work.

## Supplementary Material

dyab243_Supplementary_Data
