# Supplementary material for: Cohort Profile: The COVID-19 in Pregnancy in Scotland (COPS) dynamic cohort of pregnant women to assess effects of viral and vaccine exposures on pregnancy
Source: Int J Epidemiol. 2022 Jan 3;51(5):e245–55. doi: 10.1093/ije/dyab243 (PMC9557859; doi:10.1093/ije/dyab243)
Supplement: dyab243_Supplementary_Data [file dyab243_supplementary_data.zip › ije-2021-07-1107-File007.docx]

# **COPS technical synopsis**

The COPS cohort is constructed by integrating several disparate datasets through a series of linkage and data resolution steps (Figure 1, main manuscript).

## **Creation of pregnancy cohort**

The basis of COPS is the creation of a prospective dynamic pregnancy cohort, identifying all ongoing and completed pregnancies since 1^st^ March 2020. This cohort is constructed by interrogating population-level national datasets for records of events pertaining to pregnancy. Ongoing pregnancies are identified using antenatal booking appointment data, while completed pregnancies are identified using multiple sources, specifically general practitioner (GP) records, general acute hospital discharge records – Scottish Morbidity Record (SMR) 01; maternity hospital discharge records – Scottish Morbidity Record (SMR) 02; notification of termination data as governed by **Abortion Act Scotland (AAS)**; National Records of Scotland (NRS) statutory live birth registrations; NHS Scotland live births; and NRS statutory stillbirth registrations. These datasets capture pregnancies ending in a wide range of outcomes, specifically miscarriage, molar pregnancy, or ectopic pregnancy requiring any GP or hospital-based care, a termination of pregnancy, a live birth or a stillbirth.

The Community Health Index (CHI) number is a unique patient identifier used across all health records in Scotland, thereby enabling integration of healthcare data. All source data incorporated into COPS have been subject to a CHI-seeding process in which reported CHIs are reconciled to a contemporaneous Unique Patient Identifier (UPI) to ensure accurate linkage across sources.

1. **Event resolution**

Where multiple records within a data source pertain to the same woman, records are subject to event resolution, in which an iterative grouping procedure assigns records occurring within 83 days (i.e., less than 12 weeks) to a single healthcare event with its own universally unique identifier (UUID). Similarly, where multiple records within a data source pertain to the same woman and we have gestation information, the estimated dates of conception are used to resolve records to a single pregnancy with a UUID.

1. **Record linkage**

Following event resolution, records from each individual data source are linked together to create a comprehensive record for each pregnancy and birth. The nature of the source data dictates how this linkage is carried out. For example, live birth records from different sources are linked together using baby UPI. If baby UPI is missing, mother UPI and event date are used where possible to infer baby UPI from other sources (instances where the same baby UPI is assigned to different mothers are removed for data cleansing). This integrated dataset is again subject to the same event resolution process described above to assign records to an individual COPS event with its own UUID. Antenatal booking records are incorporated into the cohort if the booking date occurs between the earliest estimated date of conception and the latest date of end of pregnancy/delivery for that pregnancy. Where there is evidence that a pregnancy is multiple (i.e., more than one fetus), data are combined using a process that considers the outcome type, data source and birthweight to match the right data to the right fetus, so that the final cohort has the correct number of records for each pregnancy.

## **Integration of contextual information**

The cohort is then expanded to include contextual data, representing potential confounders and effect modifiers. Thus far, the contextual data in COPS includes both COVID independent characteristics (demographics, deprivation, ethnicity, smoking status, and BMI) and COVID specific characteristics (comorbidities and shielding status to create clinical vulnerability groupings, see Table 2). Deterministic linkage via CHI is used to integrate these data with the main COPS cohort. Potentially relevant mother, pregnancy and baby characteristics are also available for further analysis, having been collected at the cohort generation stage (e.g. maternal age, baby sex, baby birthweight, singleton/multiple pregnancy status).

## **Integration of data capturing exposures of primary interest**

National resources for SARS-CoV-2 infection data and COVID-19 vaccination data are incorporated into the study using CHI linkage. The COPS data infrastructure is designed such that additional diagnostic and/or exposure categories may easily be integrated as the pandemic develops and reporting demands evolve.

## **Identification of maternal, pregnancy and neonatal outcomes**

COPS seeks to capture maternal, pregnancy and neonatal outcomes and identify and quantify any association between COVID-19 infection or vaccination and these outcomes. Outcome-related data derived from various sources (specifically GP records, SMR1, SMR2, AAS, NRS & NHS livebirths, NRS stillbirths and infant death records, SICSAG, maternal deaths, linked congenital anomaly database) are assigned to each individual fetus and pregnancy using deterministic CHI linkage. Particular effort has been made to include data on early pregnancy losses (via GP records) so as to capture outcomes throughout the pregnancy timeline (Supplementary Figure 1).

## **Data consolidation**

In the generation of this cohort and the integration of data from multiple sources, the same information for the same pregnancy may be derived from alternative sources. This may be due to static data being collected multiple times (e.g., mother’s date of birth) or this may be due to data changing throughout the pregnancy (e.g., if estimated gestation and hence date of conception based on last menstrual period (LMP) at antenatal booking is subsequently updated based on more accurate dating from an ultrasound scan, reflecting the best obstetric estimate). Consolidating these data appropriately is an important step in finalising the information for each pregnancy and/or baby. To do this, derivation hierarchies were defined for COPS in order to prioritise the source of data where necessary. For example, in the case of conflicting outcomes for an individual pregnancy, termination records take precedence over live birth records, which take precedence over stillbirth records and so on. These derivation hierarchies are provided in the COPS data dictionary (available from https://github.com/Public-Health-Scotland/COPS-public).

Supplementary Figure 1. An illustration of the pregnancy outcomes captured by COPS, from conception to birth (trimester blocks are shown to scale). Note that COPS also captures neonatal outcomes up to 27 days and maternal outcomes up to 41 days post birth, though these are not shown here. ^[1]^ Data retrieval methodologies for SMR01 and GP records limits identification of events to the first trimester for each mother. ^[2]^ The minimum gestation limit in the figure is only illustrative: all live births are captured irrespective of gestation.
