# Supplementary material for: Cohort Profile: The COVID-19 in Pregnancy in Scotland (COPS) dynamic cohort of pregnant women to assess effects of viral and vaccine exposures on pregnancy
Source: Int J Epidemiol. 2022 Jan 3;51(5):e245–55. doi: 10.1093/ije/dyab243 (PMC9557859; doi:10.1093/ije/dyab243)
Supplement: dyab243_Supplementary_Data [file dyab243_supplementary_data.zip › ije-2021-07-1107-File009.docx]

**Table S1: Summary of additional data on exposures and outcomes collected in the COPS cohort study not included in Table 1**

| **Variable** | **Definition** |
| --- | --- |
| **Exposures** |  |
| Confirmed COVID-19 | Positive PCR for SARS-CoV-2 infection. Gestation in completed weeks at diagnosis. |
| Probable COVID-19 | COVID-19 recorded on a hospital admission, stillbirth, or a maternal death record (using ICD10 codes U07.1, U07.2, U07.5) in the absence of a positive PCR test.  Gestation in completed weeks at diagnosis. |
| COVID-19 vaccination | Gestation in completed weeks of each dose; dose number (first or second); type of vaccination (Pfizer, Moderna, Astra Zeneca) |
| **Pregnancy outcomes** |  |
| Any congenital anomaly | Any major congenital anomaly as defined by EUROCAT (1) (including live born babies diagnosed at <28 days of age and affected pregnancies resulting in spontaneous loss at ≥20 weeks or termination of pregnancy at any gestation) |
| Any non-genetic congenital anomaly | Any major congenital anomaly as defined by EUROCAT (1) excluding anomalies with known underlying genetic basis as per standard EUROCAT rules (including live born babies diagnosed at <28 days of age and affected pregnancies resulting in spontaneous loss at ≥20 weeks or termination of pregnancy at any gestation) |
| **Neonatal outcomes** |  |
| Small for gestational age | Small for gestational age (birthweight <10^th^ centile by World Health Organisation (WHO)-UK90 growth reference (2)) |
| Severe small for gestational age | Severely small for gestational age (birthweight <3rd centile by World Health Organisation (WHO)-UK90 growth reference (2)) |
| Microcephaly | Congenital microcephaly (i.e. present at birth) OFC on delivery record <2SD below mean for gestational age and sex by WHO-UK90 growth reference (3, 4). Babies with anencephaly excluded. |
| Severe Microcephaly | Congenital microcephaly (i.e. present at birth) OFC on delivery record <3SD below mean for gestational age and sex by WHO-UK90 growth reference (3, 4). Babies with anencephaly excluded. |
| Neonatal COVID-19 infection | Positive PCR for SARS-CoV-2 infection. Age in completed weeks (neonatal) when diagnosis made. |
| Preterm birth | Delivery at <37 completed weeks gestation following preterm prelabour rupture of membranes or spontaneous onset of labour |
| Very preterm birth | Delivery at <32 completed weeks gestation following preterm prelabour rupture of membranes or spontaneous onset of labour |
| Low Apgar score | Five-minute Apgar score less than seven |
| Very low Apgar score | Five-minute Apgar score less than four |
| Neonatal death | Death from any cause <28 days after birth |
| Extended perinatal mortality | Stillbirth or neonatal death from any cause |
| **Maternal Outcome** |  |
| Maternal Intensive Care Admission or death | ICU admission as identified on Scottish Intensive Care Society Audit Group record. Death from any cause. |
| Hypertensive disorder of pregnancy | Hypertensive disorder in pregnancy (using ICD10 codes O11, O13, O14.0, O14.1, O14.2, O14.9, O15.0, O15.1, O15.2, O15.9) |
| Deep venous thrombosis and/or pulmonary embolus | Venous thromboembolism (using ICD 10 codes I26.0, I26.9, I80.1, I80.2, I80.3, O08.2, O22.3, O87.1, O88.2, I80.8, I80.9, I81, I82.0, I82.1, I82.2, I82.3, I82.8, I82.9, O22.9, O87.9, G08, I63.6, I67.6, O22.5, O87.3) |

**References**

1. EUROCAT. EUROCAT Guide 1.4: Instruction for the registration of congenital anomalies. University of Ulster; 2013.

2. Wright CM, Booth IW, Buckler JM, Cameron N, Cole TJ, Healy MJ, et al. Growth reference charts for use in the United Kingdom. Arch Dis Child. 2002;86(1):11-4.

3. WHO. Child growth standards: head circumference-for-age; 2016. 2016

4. DeSilva M, Munoz FM, Sell E, Marshall H, Tse Kawai A, Kachikis A, et al. Congenital microcephaly: Case definition & guidelines for data collection, analysis, and presentation of safety data after maternal immunisation. Vaccine. 2017;35(48 Pt A):6472-82.
