# Supplementary figures and images for: Cohort Profile: The COVID-19 in Pregnancy in Scotland (COPS) dynamic cohort of pregnant women to assess effects of viral and vaccine exposures on pregnancy
Source: Int J Epidemiol. 2022 Jan 3;51(5):e245–55. doi: 10.1093/ije/dyab243 (PMC9557859; doi:10.1093/ije/dyab243)

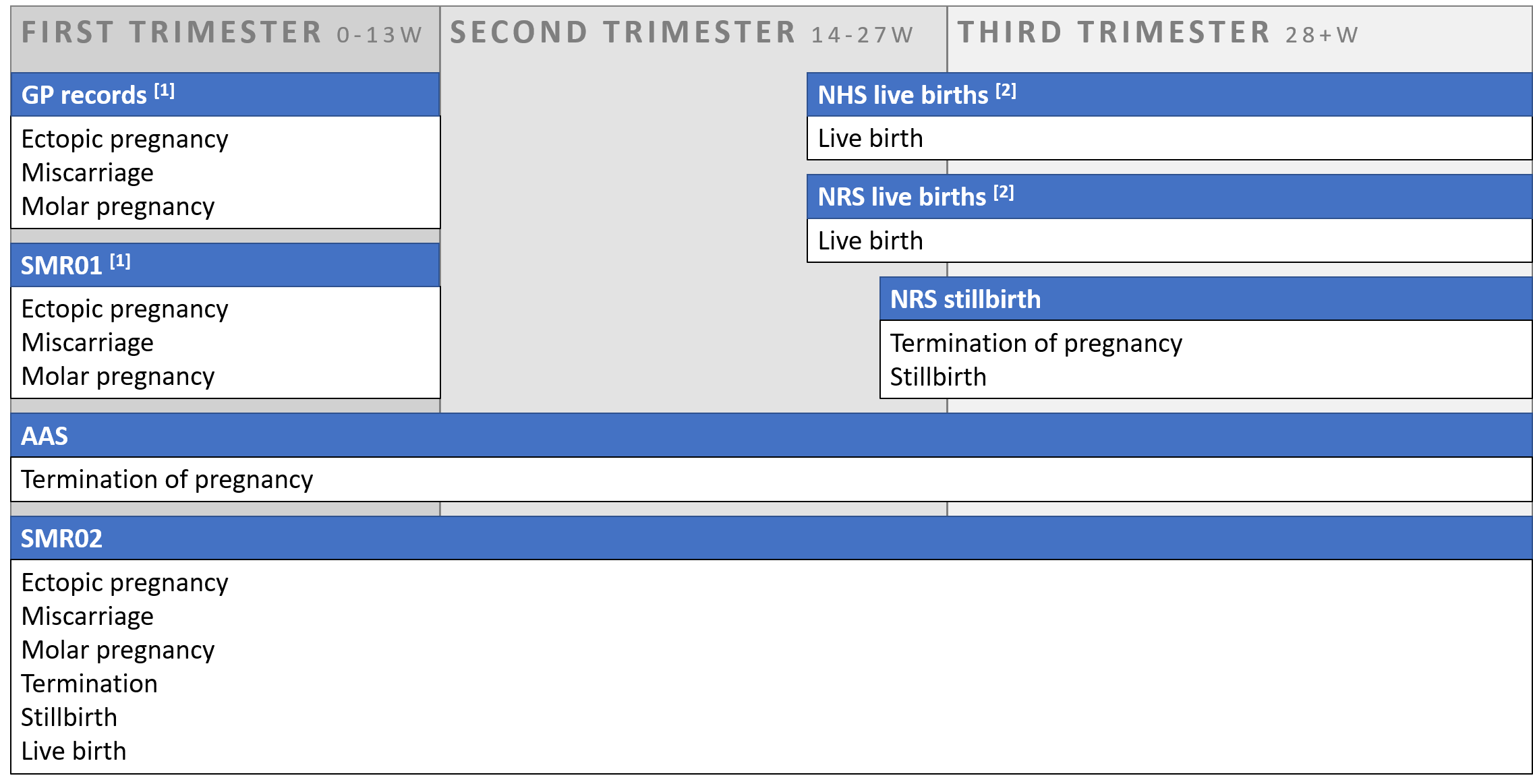

Supplement: dyab243_Supplementary_Data [file dyab243_supplementary_data.zip › ije-2021-07-1107-File008.tiff]
